# A Comparison of the Diagnostic Value of Multiorgan Point-of-care Ultrasound between High-risk and Medium-to-low-risk Pulmonary Embolism Cases

**DOI:** 10.2174/0115734056344839250120045737

**Published:** 2025-01-27

**Authors:** Weihua Wu, Zhenfei Yu, Kang Cheng, Manqiong Xie, Shunjin Fang, Jianfeng Zhu

**Affiliations:** 1 Department of Intensive Care Unit, Hangzhou TCM Hospital affiliated with Zhejiang Chinese Medical University, Hangzhou, Zhejiang Province 310007, China; 2 Department of Ultrasound, Hangzhou TCM Hospital affiliated with Zhejiang Chinese Medical University, Hangzhou, Zhejiang Province 310007, China; 3 Department of Intensive Care Unit, Huzhou Third Municipal Hospital, The Affiliated Hospital of Huzhou University, Huzhou, Zhejiang Province 313000, China

**Keywords:** Multiorgan point-of-care ultrasound, Pulmonary embolism, CT pulmonary angiography, Risk, Multiorgan PoCUS, Patients

## Abstract

**Objective:**

This study aimed to explore the diagnostic value of multiorgan (heart, lungs, blood vessels) point-of-care ultrasound (PoCUS) in patients with high-risk and medium-to-low-risk pulmonary embolism (PE).

**Methods:**

Clinical data of 92 patients with suspected PE, admitted to Hangzhou TCM Hospital affiliated with Zhejiang Chinese Medical University from July 2021 to June 2023, were retrospectively analyzed. According to hemodynamic status, patients were divided into the high-risk (n=28) and the medium-to-low-risk groups (n=64). Using computed tomography (CT) and pulmonary angiography (CTPA) as the gold standard, all patients underwent multiorgan PoCUS examination. The sensitivity, specificity, and accuracy of different methods for diagnosing PE, as well as the time difference between multiorgan PoCUS examination and CTPA, were compared. Differences in measurement values of relevant indicators in all groups were analyzed.

**Results:**

In the high-risk group of patients, CTPA identified 15 cases of PE. In contrast, the PoCUS examination confirmed PE diagnosis in 14 cases (true positive), while 10 cases were diagnosed as true negative, one case as false negative, and three cases as false positive. In the medium-to-low-risk group, CTPA identified 50 patients with PE, while multiorgan PoCUS confirmed PE diagnosis in 33 cases (true positive), and identified 9 true negative, 17 false negative, and 5 false positive PE cases. Kappa test of the consistency between the results of multiorgan PoCUS and CTPA showed that multiorgan PoCUS had higher sensitivity, negative predictive value, and accuracy in the high-risk group compared to the medium-to-low-risk group (*p*<0.05). Cohen's Kappa value of the high-risk group was 0.710, indicating moderate consistency between PoCUS and CTPA results, while Cohen's Kappa value of 0.231 for the medium and low-risk group indicated poor consistency. There was a significant difference in ultrasound parameters between the high-risk and the medium-to-low-risk group (*p*<0.05). The time required for multiorgan PoCUS in both groups was significantly shorter than that for the CTPA. There was no significant difference in the time required for PoCUS between the two groups (*p*>0.05).

**Conclusion:**

Multiorgan PoCUS has been found to have higher sensitivity and accuracy in diagnosing patients with high-risk PE compared to those with medium-to-low-risk PE, and a shorter imaging time compared to CTPA.

## 1 INTRODUCTION

Pulmonary embolism (PE) is a serious and common disease [1] that manifests as sudden difficulty in breathing, chest pain (often severe), cough, fainting, *etc* [2, 3]. In severe cases, PE may be life-threatening and lead to hypotension, shock, and cardiopulmonary failure [4]. Due to its diverse nature and non-specific clinical manifestations, accurate diagnosis of PE is crucial for treatment and prognosis.

Traditionally, computed tomography (CT) and pulmonary angiography (CTPA) are regarded as the gold standard for diagnosing PE [5]. However, despite its high sensitivity and specificity, CTPA still has some limitations, such as radiation exposure, inapplicability to patients with iodine allergy, and requirements for adequate renal function [5, 6]. With the rapid development of medical technology, echocardiography has become increasingly popular, as it can be performed at the bedside, is non-invasive, fast, radiation-free, and low-cost, and has diagnostic and exclusion value in suspected PE [7, 8]. Additionally, echocardiography is efficient for the differential diagnosis of pericardial tamponade, acute heart valve dysfunction, left heart dysfunction, and aortic dissection, which often have symptoms similar to PE [9]. However, studies have shown that direct signs detected by echocardiography only account for 2.6% of patients with PE [10]. Therefore, direct signs alone cannot be used as the main indicator for diagnosing PE by echocardiography [11]. The indirect signs of echocardiography refer to pulmonary arterial hypertension, right ventricular overload, and functional impairment caused by PE. This impairment leads to pulmonary vasoconstriction and increased right ventricular afterload, and further limits the filling of the left ventricle, resulting in a series of changes [10-12]. However, due to the unique geometric shape of the right heart, there is no separate echocardiography parameter that provides fast and reliable information about its size or function [11, 12]. This explains differences in the echocardiography diagnostic criteria of PE among different studies. Therefore, some studies have proposed multiorgan point-of-care ultrasound (PoCUS), an advanced diagnostic ultrasonography that is performed as a bedside test, to improve the accuracy of PE ultrasound diagnosis [12, 13].

This study aimed to investigate the diagnostic performance of PoCUS for PE and compare the diagnostic value of multiorgan PoCUS between high-risk and medium-to-low-risk PE cases.

## 2 MATERIALS AND METHODS

### 2.1 Patients

Clinical records of 92 patients (63 males and 29 females) with suspected PE, admitted to Hangzhou TCM Hospital affiliated with Zhejiang Chinese Medical University from July 2021 to June 2023, were retrospectively selected. The ethics committee of our hospital approved this study with no. 2021LH004 (date: November 12^th^, 2021). The principles of the Helsinki Declaration have been strictly followed for experiments involving human subjects.

### 2.2 Inclusion Criteria

The inclusion criteria were *1)* patients with suspected PE diagnosis; 2) age ≥ 18 years old; 3) underwent multiorgan PoCUS and CTPA; 4) clear image data; and 5) complete clinical data.

### 2.3 Exclusion Criteria

The exclusion criteria were 1) patients who received anticoagulant therapy before the examination; 2) cases of chronic thromboembolic pulmonary hypertension; and 3) patients with severe liver or kidney dysfunction or failure.

### 2.4 Grouping Method

According to the hemodynamic status, patients were divided into a high-risk (n=28 cases) and a medium-to-low-risk group (n=64 cases). Risk assessment of patients was as follows: if the patient presented with the main symptoms of shock or hypotension, a drop in systemic systolic blood pressure below 90mmHg (1mmHg equals 0.133kPa), or the decline that reached or exceeded 40mmHg for more than 15 minutes, and recent arrhythmias, hypovolemia or sepsis were ruled out, and the patients were classified as high-risk. All other patients were classified as medium or low-risk.

### 2.5 Multiorgan PoCUS

Imaging was done using Mindray M9 portable color ultrasound diagnostic instrument, equipped with a C5-1S convex array probe, with a frequency of 1.0~5.0 MHz, and an L12-4s linear array probe, with a frequency of 4.0~12.0 MHz.

Multiorgan PoCUS included the following:

#### 2.5.1 Transthoracic Echocardiography

**Fig. (1):**
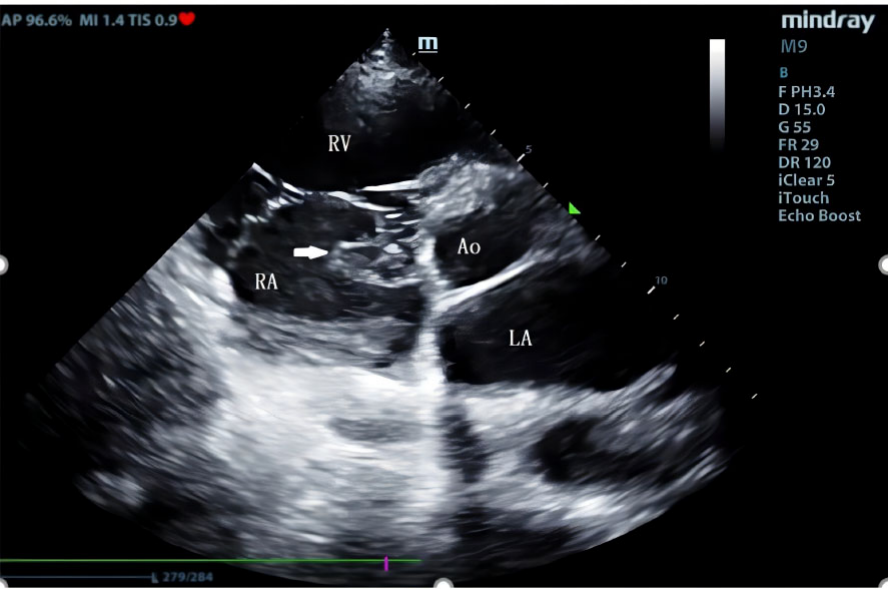
Male, 55 years old with suspected high-risk pulmonary embolism. Transthoracic echocardiography showed direct signs of thrombus in the right heart system, at the short axis level of the aorta. A thrombus was visible in the right atrium. (RV: right ventricle, RA; right atrium, LA: left atrium, Ao: aorta; arrow indicates thrombus).

Briefly, phased array probes were selected and the operating methods and diagnostic standards of the “Chinese Adult Echocardiographic Examination and Measurement Guidelines” [11] were strictly followed. The main positive signs of right heart dysfunction included the following: I. right ventricular dilation; II. reduced amplitude of free wall movement in the right ventricle (McConnell's sign); III. reduction of tricuspid annulus systolic displacement<17mm; IV. flat interventricular septum. A positive result for any of the above parameters was considered as a positive result for PE (Fig. **1**).

#### 2.5.2 Lower Limb Deep Vein Compression Ultrasound

**Fig. (2):**
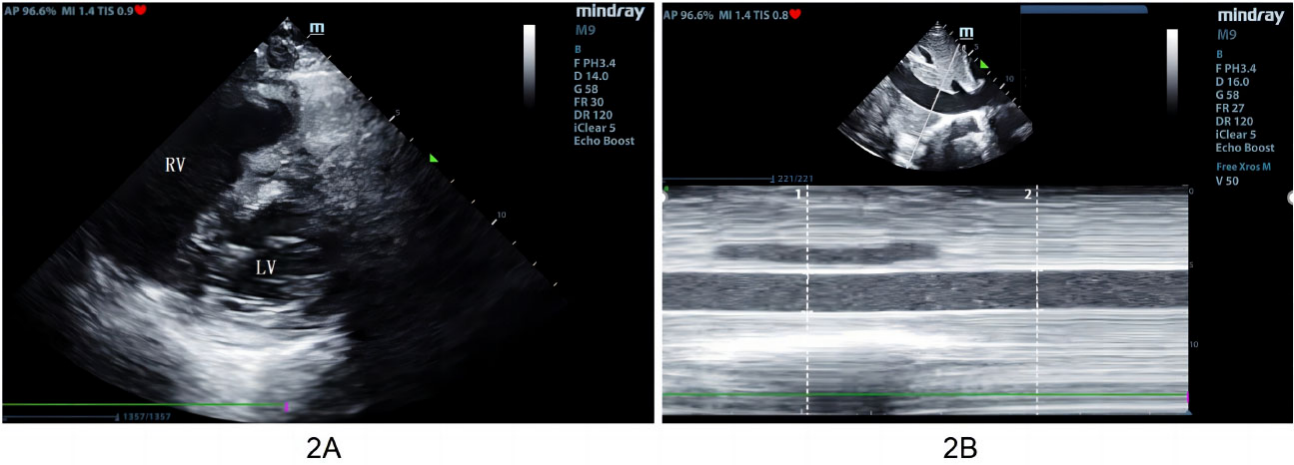
Female with age 58 years old and suspected high-risk pulmonary embolism. **A**): Transthoracic echocardiography showed false positive results, resulting in low specificity. The patient had no underlying cardiovascular or pulmonary diseases and was admitted to the hospital after cardiac arrest and resuscitation. Emergency bedside ultrasound indicated the ratio of right heart/left heart>1, and the left heart showed a “D-sign”. **B)**: Reduced movement of the right ventricular free wall (TAPSES: 1.01cm), with widened and fixed inferior vena cava. There were manifestations of right heart dysfunction, with electrocardiograms indicating a new complete right bundle branch block and widespread ST-T segment depression in V1-V6. The ultrasound determined the diagnosis of PE, but subsequent CTPA indicated a negative PE diagnosis.

**Fig. (3):**
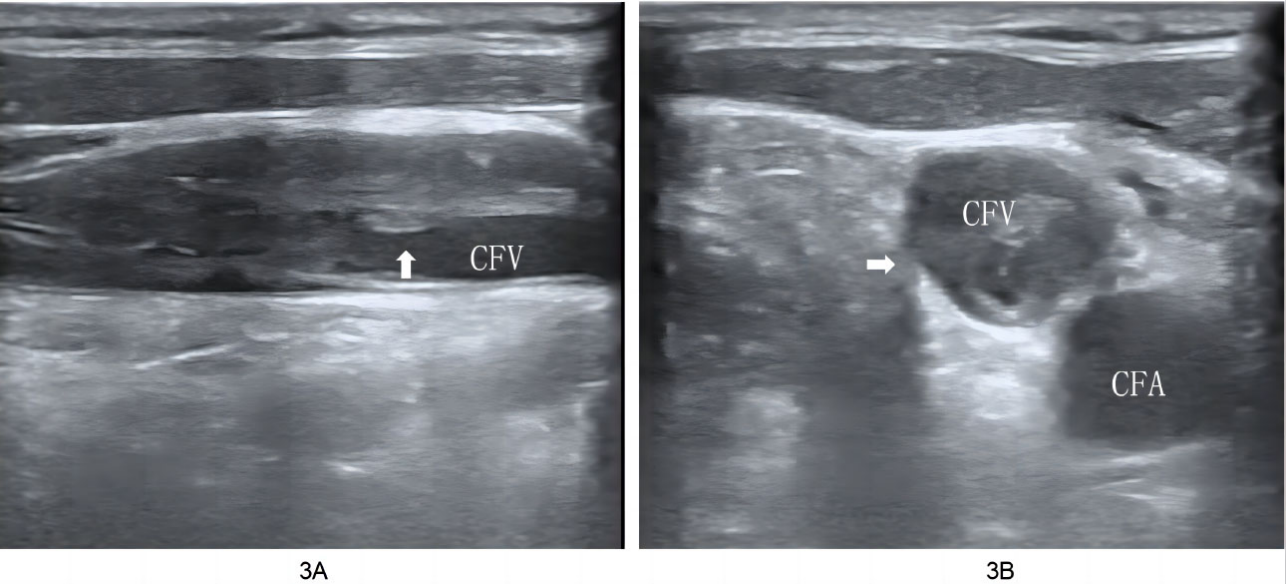
Male with age 61 years and suspected high-risk pulmonary embolism. **A)**: The white arrow indicated a common femoral vein thrombosis. **B)**: Cross-section common femoral vein thrombosis; CFV: common femoral vein; CFA: common femoral artery.

In this procedure, a linear or convex array probe was selected. The patient was placed in a supine position, and the tested lower limbs were slightly abducted or externally rotated. Intermittent compression scanning of the lower limb deep vein was performed from the groin to the lower leg transverse section. If there was a low echo, no compression, no blood flow signal, or a blood flow filling defect in the venous lumen, deep vein thrombosis (DVT) formation was indicated as PE positive (Figs. **2** and **3**).

#### 2.5.3 Pulmonary Ultrasound

**Fig. (4):**
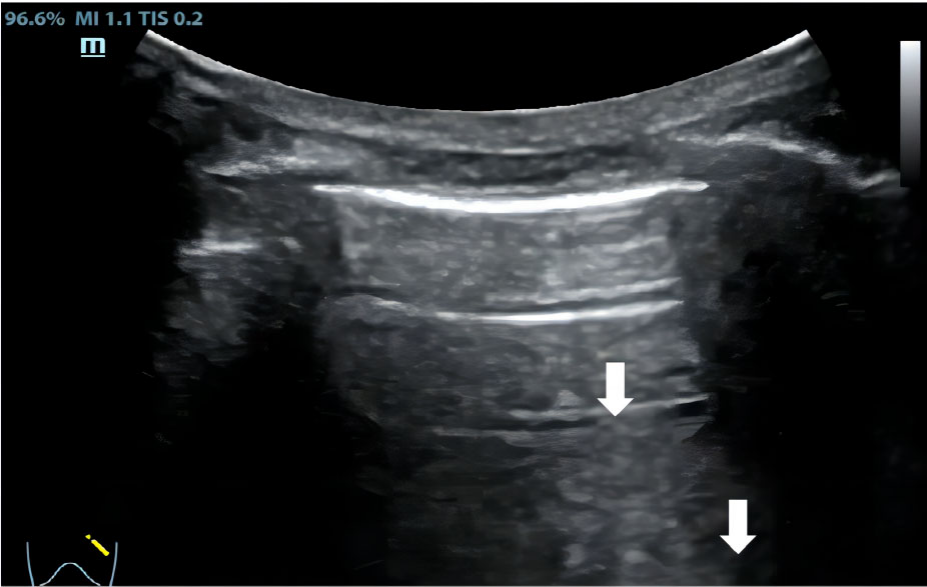
Female aged 63 years old with suspected high-risk pulmonary embolism. In pulmonary ultrasound, the white arrow indicated the A-line. Wedge-shaped solid hypoechogenicity was discovered in subpleural lung tissue.

Convex or linear array probes were selected, and the patient was placed in a supine or seated position. The anterior chest, lateral chest, and posterior chest were examined sequentially. If lung tissue under the pleura was triangular, wedge-shaped, or circular with solid hypoechogenicity, it was considered positive for PE (Fig. **4**).

Two physicians with extensive experience and ultrasound qualifications independently conducted bedside multiorgan (heart, lungs, blood vessels) PoCUS and made separate diagnoses. The ultrasound diagnosis was established only if both physicians reached a consensus. In cases of disagreement, a third physician with ultrasound qualifications conducted another ultrasound examination and reached the same conclusion.

### 2.6 Indicators

The following indicators were taken into account: 1) clinical data of patients; 2) cardiac indicators, including the presence of blood clots in the right heart and main pulmonary artery; important measurement parameters, such as right/left ventricular aspect ratio, main pulmonary artery diameter, peak tricuspid regurgitation pressure difference, pulmonary artery pressure, inferior vena cava diameter, *etc*.

### 2.7 Statistical Analysis

SPSS version 26.0 (IBM Corp, Armonk, NY, USA) was used for data analysis. For categorical variables, frequency distribution was provided and expressed as the percentage. A Chi-square test was used to compare categorical variables between the two groups. The diagnostic consistency between multiorgan PoCUS and CTPA methods was analyzed using the Kappa test. Data with normal distribution are represented by mean ± standard deviation (SD), and an independent sample t-test was used for inter-group comparison. *p*<0.05 was considered statistically significant.

## 3 RESULTS

Among the 92 patients included, 63 were male and 29 were female. The age range was 48-82 years old, with an average age of 64.4±7.7 years old. There were 28 cases in the high-risk group and 64 cases in the medium-to-low-risk group; There was no statistically significant difference in baseline data between the two groups of patients (*p*>0.05) (Table **1**).

Based on the CTPA assessment, PE diagnosis was confirmed in 15 of 28 high-risk patients. In contrast, multiorgan PoCUS classified 14 out of 28 high-risk patients as true positive for PE, while 10 cases were true negative, 1 case was false negative, and 3 cases were false positive (Table **2**).

Among 64 medium-to-low-risk PE patients, 50 were diagnosed with PE after CTPA assessment. Multiorgan PoCUS classified 33 out of 64 patients in the medium-to-low-risk group as true positive. Nine patients were classified as true negative, 17 as false negative, and 5 as false positive (Table **3**).

The Kappa test was then used to analyze the consistency between the results of multiorgan PoCUS and CTPA in the two groups. The results showed that multiorgan PoCUS had high sensitivity and negative predictive value, and the accuracy of the method in the high-risk group was significantly higher compared to the medium to low-risk group (*p*<0.05). The Cohen's Kappa value of the high-risk group was 0.710, indicating moderate consistency between the PoCUS and CTPA methods. However, the Cohen's Kappa value for the medium-to-low-risk group was 0.231, indicating poor consistency (Table **4**). The right/left ventricular aspect ratio, main pulmonary artery diameter, peak tricuspid regurgitation pressure difference, pulmonary artery pressure, and inferior vena cava diameter values in the high-risk group were all higher than those in the medium-to-low-risk group. The time of pulmonary artery blood flow acceleration, inferior vena cava collapse rate, and tricuspid annular systolic displacement were significantly lower in the high-risk group compared to the medium-to-low-risk group (*p*<0.05) (Table **5**). Multiorgan PoCUS procedure time for both groups was significantly shorter than that of the CTPA (*p*<0.05). There was no significant difference in the time required for multiorgan PoCUS between the two groups (*p*>0.05) (Table **6**).

## 4 DISCUSSION

This study showed multiorgan PoCUS to have higher sensitivity and accuracy in diagnosing patients with high-risk PE compared to those with medium-to-low-risk PE, and a shorter imaging time compared to CTPA. The results indicated that compared to patients with medium-to-low-risk PE, multiorgan PoCUS can better identify and rule out high-risk patients with PE. Based on Cohen's Kappa values, there was a moderate consistency between multiorgan PoCUS and CTPA results in the high-risk group. This observation has been found to be in agreement with the findings of Bailis *et al*. [14]. Since cardiac ultrasound can evaluate the structure and function of the heart, inferior vena cava ultrasound can evaluate the morphology, function, and blood flow of the inferior vena cava and its surrounding structures, and pulmonary ultrasound can evaluate lung structure and hemodynamics [13, 15], it is plausible that the combined application of these three ultrasound examinations can comprehensively evaluate the cardiovascular system of patients with PE, helping to identify the causes and related complications of PE [13, 15, 16]. Additionally, the diagnosis of PE requires a comprehensive examination of multiorgan PoCUS results. A combination of cardiac, vena cava, and pulmonary ultrasounds allows to simultaneously evaluate right ventricular function and pulmonary artery pressure, detect thrombosis in the inferior vena cava and femoral vein, and assess the blood flow of pulmonary arteries and pulmonary artery branches. Ultimately, this can improve the sensitivity and specificity of PE diagnosis, as well as its severity [16-18]. Furthermore, cardiac, vena cava, and pulmonary ultrasounds are all real-time examination methods that can be performed at the bedside or in an emergency setting. This allows clinicians to immediately assess ultrasound images, evaluate changes in the patient's condition in a timely manner, and guide emergency treatment and intervention measures [19, 20]. Finally, cardiac, vena cava, and pulmonary ultrasounds are non-invasive and do not require injection of contrast agents or surgery [17, 21].

Our results showed poor consistency between multiorgan PoCUS results and CTPA in the medium and low-risk groups, with lower Kappa values. This may be due to the overall low risk of patients in this group, making the diagnosis of PE more difficult. This study also showed that in the high-risk group, the right/left ventricular aspect ratio, main pulmonary artery diameter, peak pressure difference in mitral regurgitation, pulmonary artery pressure, and inferior vena cava diameter were higher than those in the medium-to-low-risk group. These parameters reflected the hemodynamic state of the pulmonary artery system and the load situation of the heart. In the high-risk group, pulmonary artery blood flow was found to be subjected to significant pressure and volume load, possibly due to PE-associated pulmonary hypertension and increased right heart load [21]. In addition, in the high-risk group, the acceleration time of pulmonary artery blood flow, the rate of inferior vena cava collapse, and the displacement of tricuspid annulus during closure were lower than those in the medium-to-low-risk group. The above parameters reflected the efficiency of heart function and hemodynamic regulation. In the high-risk group, PE caused increased blood flow resistance and right heart load, leading to impaired cardiac function and hemodynamic regulation [22]. The results of this study suggested patients in the high-risk group to have significant abnormalities in pulmonary artery flow and cardiac function compared to the medium-to-low-risk group. We may speculate that these differences could be due to hemodynamic changes caused by PE and insufficient adaptive response of the heart.

Multiorgan PoCUS may allow clinicians to better evaluate PE severity, cardiac function status, and prognostic risks in patients, as well as timely initiate appropriate treatment measures [17]. The time needed to perform and obtain results from the two sets of CTPAs was significantly higher than that of multiorgan PoCUS. This has been found to be consistent with the observation of Chen *et al*. [23]. CTPA procedure requires intravenous injection of contrast agent, waiting for image acquisition and analysis, *etc*. In contrast, during multiorgan PoCUS, no injection of contrast agents or waiting for image processing are needed. Our results also indicated the duration of CTPA to be significantly longer in the high-risk group of patients, compared to the medium-to-low-risk group. We may speculate that high-risk patients generally present with more severe symptoms and may require more complex image acquisition and analysis to evaluate the course and extent of thrombosis. Our results indicated CTPA to require overall more time to obtain results compared to multiorgan PoCUS. Therefore, in high-risk patients who urgently need rapid diagnosis and treatment, PoCUS may be an effective screening tool that could provide the required assessment in the most efficient and timely manner. Multiorgan PoCUS method can achieve a more comprehensive and effective diagnosis of PE and may be recommended in general practice.

This study has involved some limitations. Firstly, this was a single-center retrospective study. Incomplete medical records and bias in recalling medical history may have led to selection bias. Secondly, ultrasound indicators may have been influenced by human or technical factors. Thirdly, the specific location of PE and the patient's prognosis were not analyzed. Fourthly, CTPA and multiorgan PoCUS procedure time may be quite different according to each institute. Further high-quality research is needed to validate our conclusions, and we also propose to evaluate the outcomes of PE patients with findings obtained in the current study by PoCUS in future studies.

## CONCLUSION

Multiorgan PoCUS has been found to have higher sensitivity and accuracy in diagnosing high-risk PE patients compared to those with medium-to-low-risk PE, and to be associated with shorter procedure time compared to CTPA.

## Figures and Tables

**Table 1:** Comparison of baseline data between two groups of patients.

Baseline Data	High Risk Group (*n*=28)	Middle to Low Risk Group (*n*=64)	*X^2^/t*	*p*
Male (yes), n(%)	17 (60.7)	46 (71.9)	1.124	0.289
Age (year), mean±SD	63.4±7.0	64.9±7.9	-0.831	0.408
Time from onset to hospitalization (day)	7.2±2.1	7.3±2.6	-0.209	0.835
Hypertension (yes), n(%)	7 (25.0)	15 (23.4)	0.026	0.872
Diabetes (yes), n(%)	8 (28.6)	13 (20.3)	0.252	0.616
Coronary heart disease (yes), n(%)	4 (14.3)	6 (9.4)	0.485	0.486
History of smoking (yes), n(%)	9 (32.1)	18 (28.1)	0.152	0.697
History of drinking (yes), n(%)	7 (25.0)	22 (34.4)	0.793	0.373

**Note: **SD-standard deviation.

**Table 2:** Comparison of results of different examination methods in high-risk groups.

Multi Organ PoCUS	CTPA		Total
	Positive	Negative	
Positive	14	3	17
Negative	1	10	11
Total	15	13	28

**Note: **PoCUS-point-of-care ultrasound; CTPA-computed tomography pulmonary angiography.

**Table 3:** Comparison of results of different examination methods in middle-low risk group.

Multi Organ PoCUS	CTPA		Total
	Positive	Negative	
Positive	33	5	38
Negative	17	9	26
Total	50	14	64

**Note: **PoCUS-point-of-care ultrasound; CTPA-computed tomography pulmonary angiography.

**Table 4:** Comparison of ultrasound diagnostic value between the two groups.

Group	Diagnostic Accuracy	Sensitivity	Specificity	Positive Prediction Rate	Negative Prediction Rate	*Cohen´s Kappa*
High risk group (*n*=28)	85.71	93.33	76.92	82.35	90.91	0.710
Middle to low risk group (*n*=64)	65.63	66.00	64.29	86.84	34.62	0.231

**Note:** *Kappa*≥0.75 indicates excellent consistency; 0.75 > *Kappa*≥0.4 indicates moderately consistent; *Kappa* < 0.4 indicates the consistency difference.

**Table 5:** Comparison of the measurement values of relevant indicators between the two groups.

Index	High Risk Group (*n*=28)	Middle and Low Risk Group (*n*=64)	*t*	*p*
Right ventricular/left ventricular transverse diameter ratio	1.09±0.28	0.69±0.14	7.105	0.000
Main pulmonary artery diameter /mm	28.18±2.87	23.41±5.21	5.633	0.000
Pulmonary artery blood flow acceleration time /ms	66.50±5.17	78.41±5.55	-9.661	0.000
Peak pressure difference of tricuspid regurgitation /mmHg	45.39±4.53	33.80±8.32	8.608	0.000
Pulmonary artery pressure /mmHg	51.25±6.77	38.92±7.45	7.502	0.000
Internal diameter of inferior vena cava /mm	24.50±4.26	20.05±3.87	4.920	0.000
Collapse rate of inferior vena cava /%	51.21±4.18	56.56±3.13	-6.787	0.000
Tricuspid ring systolic displacement /mm	16.21±2.10	19.56±1.55	-8.524	0.000

**Table 6:** Comparison of time required for the procedure (minutes).

Group	CTPA	Multi Organ PoCUS	*t*	*p*
High risk group (n=28)	40.86±9.19	13.71±3.17	13.125	0.000
Middle to low risk group (n=64)	36.63±7.53	13.20±2.84	23.731	0.000
*t*	2.317	0.767	-	-
*p*	0.023	0.445	-	-

**Note: **PoCUS-point-of-care ultrasound; CTPA-computed tomography pulmonary angiography.

## Data Availability

The authors confirm that the data supporting the findings of this research are available within the article.

## LIST OF ABBREVIATIONS

PoCUS: Point-of-care ultrasound
PE: Pulmonary embolism
CT: Computed tomography

## STANDARDS OF REPORTING

STROBE guidelines were followed.
